# Pharmacokinetics and tissue residue of enrofloxacin in healthy, *Eimeria*-infected broiler chickens and those pre-treated with amprolium and toltrazuril

**DOI:** 10.1080/23144599.2020.1765720

**Published:** 2020-06-01

**Authors:** M. Atef, H. A. El-Banna, H.Y. Elzorba, A. M. Soliman

**Affiliations:** Department of Pharmacology, Faculty of Veterinary Medicine, Cairo University, Cairo, Egypt

**Keywords:** Disposition, enrofloxacin, HPLC, pharmacokinetics, tissue residues

## Abstract

The pharmacokinetics of enrofloxacin was compared in healthy chickens, *Eimeria* infected chickens and in *Eimeria* infected chickens pre-treated with amprolium or toltrazuril following a single IV and oral administration at dose 10 mg/kg. The blood samples were taken after administration at different time intervals (5 min to 24 hours) to determine the pharmacokinetic parameters of enrofloxacin. The different concentrations of enrofloxacin were determined by using HPLC assay method. Serum concentrations versus time were analysed by a non-compartmental method. The results explored a significant decrease in serum concentrations of enrofloxacin at different time intervals and a significant change in pharmacokinetic profiles in *Eimeria* infected chickens compared with those values in healthy chickens whereas, amprolium improves these values. Toltrazuril leads to a significant decrease in enrofloxacin concentrations compared with infected non-treated chickens. Multiple-dose study revealed a longer withdrawal period of enrofloxacin in infected non-treated and infected chickens pre-treated with amprolium compared with the healthy group.

## 1. Introduction

Fluoroquinolones or fluorinated quinolones are developed chemotherapeutic agents characterized by good absorption, good tissue and cell penetration and relatively long half-lives [1–3]. Enrofloxacin is one of the most commonly used fluoroquinolones. It has a bactericidal effect against Gram-negative bacteria, *Mycoplasma* and aerobic, anaerobic bacteria including strains resistant to many other antimicrobial agents [4]. Enrofloxacin is indicated clinically for the treatment of gastroenteric, respiratory, urogenital and skin infections in pigs, calves, cattle, and poultry [5,6]. Several kinds of research were carried out for pharmacokinetics pattern of enrofloxacin in different species including pigs [7], rabbits [8], sheep [9,10], goats [11], horses [12,13], poultry [5,6,14,15], turkey [16] and Japanese quail [4] under normal conditions. Moreover, it has been reported that pharmacokinetics of enrofloxacin is altered when concurrent administration with albendazole in calves [17].

Coccidiosis is a particularly dangerous disease in the poultry industry where chickens are raised on the floor [18]. Anti-coccidial drugs of many different types have been the dominant means to prevent and control the coccidiosis [19]. Toltrazuril is a symmetrical triazinetrione compound and it has coccidiocidal action against all intracellular developmental stages including those of schizogony and gametogony [20,21]. Toltrazuril gave better protection against coccidiosis when compared with halofuginone in drinking water when administered 4 and 5 days after inoculation [22].

Accordingly, this study was conducted to compare the pharmacokinetics of enrofloxacin in chickens (healthy, infected non-treated, infected pre-treated with amprolium and toltrazuril) after a single IV and oral administration and to determine tissue distribution and withdrawal time after a multiple oral doses of enrofloxacin for 5 consecutive days.

## 2. Material and methods

### 2.1. Ethical statement

The current study protocol was approved by the Institutional Animal Care and Use Committee in the Faculty of Veterinary Medicine, Cairo University (Protocol number 2211201809/2019).

### 2.2. Drugs

#### 2.2.1. Enrofloxacin

It was obtained from Pharma-Swede Company – Egypt as oral solution (10%) and injectable solution (5%) under trade name Avitryl®.

#### 2.2.2. Amprolium

It was obtained from Adwia Company – Egypt as white powder 20% under trade name Amprolium 20%®.

#### 2.2.3. Toltrazuril

It was obtained as 2.5% oral solution from Pharma-Swede Company – Egypt under trade name Tolacox**®**.

### 2.3. Birds and experimental design

Seventy-two healthy broiler chickens of nearly 4 weeks age and 1000–1300 gram weight were used. Chickens were obtained from a private poultry farm in Cairo – Egypt. The birds were kept under good hygienic measures; water and feed were offered *ad-libitum*. Ration was free from any medications or feed additives. Birds were kept for 2 weeks before starting the experiments to ensure that they are free from any antibacterial drugs or any disease or coccidiosis.

Chickens were divided into four groups; Group (A) includes 18 normal healthy chickens. Group (B) includes 18 experimentally infected broiler chickens with *Eimeria* spp (non-treated) for studying the pharmacokinetics, tissue distribution and withdrawal period of enrofloxacin in infected non-treated birds. Group (C) includes 18 experimentally infected broiler chickens with *Eimeria* spp (pre-treated with amprolium 240 ppm for 5 consecutive days before enrofloxacin administration). Group (D) includes 18 experimentally infected broiler chickens with *Eimeria* spp (pre-treated with toltrazuril 25 ppm for 2 consecutive days before enrofloxacin administration).

The following experiments were performed on groups A, B, C and D.

#### 2.3.1. Experiment 1

Study the pharmacokinetic profiles of enrofloxacin following a single IV administration of enrofloxacin (10 mg/kg b.wt.) in the right brachial wing vein.

#### 2.3.2. Experiment 2

Study the pharmacokinetic profiles of enrofloxacin following a single oral administration of enrofloxacin (10 mg/kg b.wt.) and these chickens were used for experiment 3.

#### 2.3.3. Experiment 3

Study the tissue distribution and withdrawal time of enrofloxacin following oral administration of enrofloxacin (10 mg/kg b.wt. once daily for 5 consecutive days).

Three chickens were slaughtered after 2 hours and 1, 3, 5,7,10 days following the last oral dose.

Samples from blood, heart, lung, liver, kidney, spleen, brain, thigh muscles and breast muscles were taken from slaughtered chickens for assaying of enrofloxacin concentration.

### 2.4. Propagation, preparation of the oocysts and experimental infection

Eight-days old chicken (free from coccidia reared on wire cages) was inoculated with sporulated *Eimeria* mixed oocyst suspension 15,000 sporulated oocyst/ml suspension/chick. The infected caeca and intestine were collected on the 7th day post-infection and prepared. The different species of *Eimeria* present in the used inoculums were identified according to the difference in size (after measuring 100 oocysts) from each size group [23]. The collected sporulated oocysts were used for the induction of experimental infection of 20-days-old chicken.

The daily output of *Eimeria* oocysts in droppings of infected birds was counted from the 5th day to the 11th day post-infection using the McMaster technique as described by Velkers et.al. [23]. Post-mortum examination for determination of intestinal lesions was performed.

### 2.5. Blood sampling and analytical procedure

Blood samples were taken from the left-wing vein at 5,10,15,30, 45 minutes and 1, 2, 4, 6, 8, 12, 24 hours after IV injection and at 15, 30 minutes and 1, 2, 4, 6, 8, 12, 24 hours after oral administration of enrofloxacin (10 mg/kg b.wt.) for determination of pharmacokinetics, tissue distribution and withdrawal period of enrofloxacin in control healthy and infected broiler chickens either infected non-treated or treated with amprolium or toltrazuril. Serum concentrations of enrofloxacin were measured by using HPLC according to the method described by El-Banna et.al. [24]. The HPLC system included: a TSP unit equipped with one pump; TST-P1000 unit equipped with a TSP-600LP UV-Vis variable lamp diode array and TSP fluorescent detector; an Altex-210A manual injector with 50 microlitre sample loop; and a Chromo-Quest computing integrator software. Kromasil C18 column; stainless steel (250 × 4.6 mm ID, particle size 10 µm) at a flow rate, 1.5 ml/mint; wavelength, 276 nm for the fluorescent detector. The retention time for enrofloxacin was 4.1 minutes. The limit of detection was 0.06 µg/ml, while the limit of quantification was 0.1 µg/ml. The intra- and inter-day assay coefficient of variation of enrofloxacin were ˂4.2 and ˂5.1, respectively, and the recovery of enrofloxacin using this method reaches 96%.

### 2.6. Pharmacokinetic and statistical analysis

Serum concentrations of enrofloxacin after IV and oral administrations were subjected to a non-compartmental software program (WinNonlin® software, version 5.2, Phar sight Corporation, NC, USA). The area under the serum concentration vs. time curve (AUC_0-∞_) was calculated using the linear trapezoidal rule with extrapolation to infinity. Maximum serum concentration (C_max_) and the corresponding peak time (T_max_) were determined from the data by the software program the inspection of the individual drug serum concentration-time profiles. The slope of the terminal phase of the time–concentration curve was determined by linear regression and converted to an elimination half-life (T_1/2λ_z). Data were expressed as mean ± S.E. and were statistically analysed using analysis of variance (ANOVA). Mean comparisons were performed using Tukey’s test. The differences were considered significant when p < 0.05. These calculations were performed using Prism 5.0 (GraphPad Software, San Diego, CA, USA).

## 3. Results

**Figure 1.**
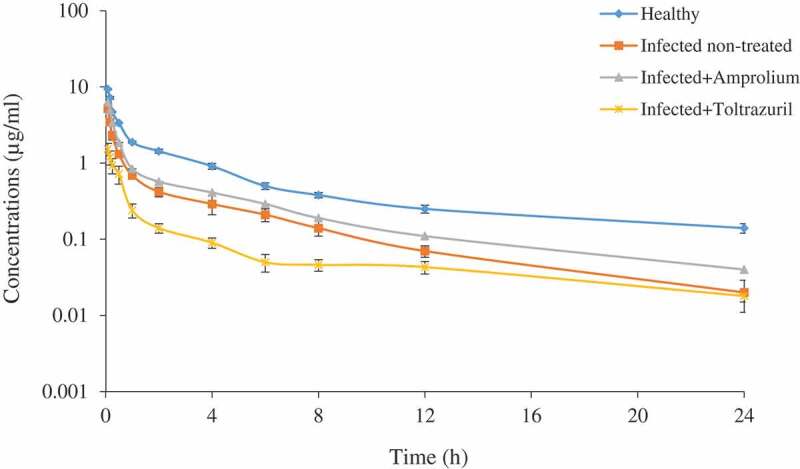
Serum concentrations of enrofloxacin (µg/ml) in healthy chicken, *Eimeria* infected non-treated and *Eimeria* coccidia infected chicken treated with either amprolium or toltrazuril after a single intravenous dose of 10 mg/kg b.wt. Mean ± SE (n = 6)

**Figure 2.**
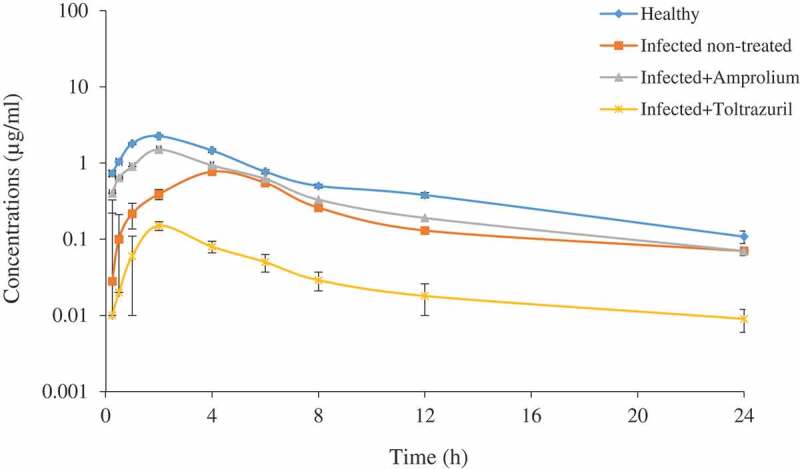
Serum concentrations of enrofloxacin (µg/ml) in healthy chicken, *Eimeria* infected non-treated and *Eimeria* infected chicken treated with either amprolium or toltrazuril after a single oral dose of 10 mg/kg b.wt. Mean ± SE (n = 6)

No signs of toxicity or any physical abnormalities were observed on the experimental chickens after enrofloxacin administration. The serum concentration–time curves of enrofloxacin following IV administration, and the values of calculated pharmacokinetic data, are illustrated in Figure 1 and Table 1, respectively. After IV administration, enrofloxacin distributed with a Vdss of 34.00 ± 3.67 L/kg in infected pre-treated with toltrazuril group compared with normal healthy group 5.07 ± 0.18 L/kg. The serum concentration–time curves of enrofloxacin following oral administration, and the values of calculated pharmacokinetic data, are illustrated in Figure 2 and Table 2, respectively. The peak of enrofloxacin serum concentration C_max_ was higher in the normal healthy group (2.06 μg/ml) achieved at 1.84 hours compared with the other groups. A higher value of systemic bioavailability F% was calculated in healthy chickens (77.3%) compared with values recorded in infected non-treated birds (54.7%). There is a significant increase of bioavailability in the infected pre-treated group with amprolium (63.5%) compared with infected non-treated birds. On contrary, there is a significant decrease of bioavailability in infected birds pre-treated with toltrazuril (44.16%) compared with infected non-treated birds.

**Table 1.** Pharmacokinetic parameters of enrofloxacin in healthy chickens, *Eimeria* infected non-treated and *Eimeria* infected chickens pre-treated with either amprolium or toltrazuril following a single intravenous dose of (10 mg/kg b.wt.)

Parameter	Unit	Healthy	Infected non-treated	Infected treated with amprolium	Infected treated with toltrazuril
λz	h^−1^	2.8 ± 0.09	3.04 ± 0.226	3.03 ± 0.1	2.26 ± 0.16^x^
T_1/2λz_	h	6.9 ± 0.5	5.21 ± 0.32*	5.87 ± 0.36	7.98 ± 0.2^xx^
V_C_	L/kg	0.89 ± 0.03	1.6 ± 0.17**	1.32 ± 0.14	4.77 ± 0.26^xxx^
Vd_ss_	L/kg	5.07 ± 0.18	9.23 ± 1.5**	7.7 ± 0.59	34.00 ± 3.67^xxx^
CPº	µg/ml	10.8 ± 0.3	6.176.±0.52***	7.7 ± 0.55	1.95 ± 0.23^xxx^
Clb_tot_	L/h/kg	0.66 ± 0.04	1.93 ± 0.18***	1.3 ± 0.07^x^	5.76 ± 1.113^xx^
AUC_0-∞_	hr×μg/ml	15.2 ± 1.08	5.55 ± 0.49***	7.38 ± 0.61^x^	2.07 ± 0.4^xxx^
AUMC	hr×hr×μg/ml	117.25 ± 12.6	24.71 ± 3.2***	44.03 ± 5.8^xxx^	16.11 ± 1.5^xxx^
MRT_0-∞_	h	7.71 ± 0.9	4.45 ± 0.7*	5.96 ± 0.1^x^	7.78 ± 0.9

Abbreviations: λz: elimination phase constant; T_1/2_λz: half-life of elimination phase; Vdss: volume of distribution after IV; CP^0^: zero-concentration; Clb_tot_: total body clearance; AUC: area under the serum concentration–time curve; AUMC: area under the first-moment curve; MRT_0–∞_: mean residence time.

Healthy compared with Infected non-treated.

*Sig. at P ≤ 0.05 **Sig. at P ≤ 0.01 ***Sig. at P ≤ 0.01.

Infected pre-treated with amprolium and Infected pre-treated with toltrazuril compared with Infected non-treated.

^x^Sig. at P ≤ 0.05, ^xx^Sig. at P ≤ 0.01, ^xxx^Sig. at P ≤ 0.001.

Mean ± SE (n = 6).

**Table 2.** Pharmacokinetic parameters of enrofloxacin in healthy chickens, *Eimeria* infected non-treated chickens and *Eimeria* infected chicken pre-treated with either amprolium or toltrazuril following a single oral administration of (10 mg/kg b.wt.)

Parameter	Unit	Healthy	Infected non-treated	Infected treated with amprolium	Infected treated with toltrazuril
λz	h^−1^	0.23 ± 0.006	0.25 ± 0.014	0.34 ± 0.05	0.335 ± 0.02
T_1/2λz_	h	2.97 ± 0.07	2.7 ± 0.21	1.86 ± 0.09^xx^	2.08 ± 0.15^x^
C_max_	µg/ml	2.06 ± 0.06	0.6 ± 0.078***	1.3 ± 0.12^xx^	0.12 ± 0.013^xxx^
T_max_	h	1.84 ± 0.06	3.6 ± 0.17***	2.05 ± 0.04^xxx^	2.29 ± 0.04
AUC_0-∞_	hr.μg/ml	11.67 ± 0.71	3.01 ± 0.039***	4.66 ± 0.38^xx^	0.85 ± 0.12^xxx^
AUMC_0-∞_	hr.hr.μg/ml	30.0 ± 1.7	37.31 ± 1.2	5.58 ± 0.06^xxx^	1.5 ± 0.02^xxx^
MRT_0-∞_	h	2.57 ± 0.09	12.40 ± 0.8**	1.20 ± 0.01	1.76 ± 0.05
F	%	77.3 ± 3.53	54.7 ± 2.04***	63.5 ± 4.037	44.16 ± 4.9^xx^
R.F	%	––	25.8 ± 2.04***	39.93 ± 1.97	7.28 ± 0.88^xxx^

Abbreviations: λz: elimination phase constant; T_1/2_λz: half-life of elimination phase; *C*_max_: peak serum concentration; AUC: area under the serum concentration–time curve; AUMC: area under the first-moment curve; MRT_0–∞_: mean residence time; F: systemic bioavailability; R.F: Relative bioavailability.

Healthy compared with Infected non-treated.

*Sig. at P ≤ 0.05 **Sig. at P ≤ 0.01 ***Sig. at P ≤ 0.001.

Infected pre-treated with amprolium and Infected pre-treated with toltrazuril compared with Infected non-treated.

^x^Sig. at P ≤ 0.05, ^xx^Sig. at P ≤ 0.01, ^xxx^Sig. at P ≤ 0.01.

Mean ± SE (n = 6).

Tissue distribution and withdrawal time of enrofloxacin were recorded and revealed a wide distribution of enrofloxacin in tested tissues (serum, liver, kidney, lung, brain, breast muscles, thigh muscles, spleen and heart) in all tested groups. Enrofloxacin was still detected in serum at the 3rd day after oral administration in healthy broiler chickens, while it was detected in tissues of healthy broilers on the 5th day after stopping of the drug medication, while for 7th day in tissues of infected birds, 10th day in the tissue of infected birds pre-treated with amprolium and only for 5 days in infected pre-treated with toltrazuril (Table 3). The highest concentration was detected in the liver and kidney while the lowest value was determined in the brain.

**Table 3.** Tissue concentrations of enrofloxacin (µg/gm) in chickens after multiple oral doses of 10 mg/kg b.wt. once daily for 5 consecutive days

	Organs									
Time of slaughter	Chicken groups	Serum	Liver	Kidney	Lung	Brain	Breast muscle	Thigh muscles	Spleen	Heart
2 h	Healthy	2.2	2.7	1.95	1.54	0.8	0.68	0.38	1.53	0.76
	*Eimeria* infected non-treated	2.46	2.48	1.82	2.07	0.5	2.12	1.45	1.35	0.8
	*Eimeria* infected treated (Amprolium)	2.16	5.25	3.3	3.1	1.85	2.2	2.07	3.63	2.4
	*Eimeria* infected treated (Toltrazuril)	0.22	1.79	1.7	1.36	0.47	1.6	1.53	1.2	1.59
1 day	Healthy	0.35	1.09	1.29	0.76	0.4	0.24	0.19	0.76	0.33
	Eimeria infected non-treated	0.36	1.12	0.37	0.32	0.04	0.35	0.4	0.39	0.4
	*Eimeria* infected treated (Amprolium)	0.7	0.68	0.63	0.3	0.11	0.62	0.48	0.54	0.17
	*Eimeria* infected treated (Toltrazuril)	0.03	0.03	0.02	0.015	0.004	0.072	0.21	0.007	0.023
3 day	Healthy	0.09	0.53	0.17	0.09	0.03	0.07	0.04	0.19	0.056
	*Eimeria* infected non-treated	0.03	0.055	0.017	0.025	0.015	0.125	0.35	0.04	0.05
	*Eimeria* infected treated (Amprolium)	0.03	0.15	0.015	0.051	0.009	0.12	0.11	0.11	0.016
	*Eimeria* infected treated (Toltrazuril)	–	0.015	0.009	0.009	0.003	0.029	0.014	0.005	0.0075
5 day	Healthy	–	0.3	–	0.004	–	0.019	0.029	–	–
	*Eimeria* infected non-treated	0.012	0.027	0.005	0.038	0.0085	0.01	0.008	0.011	0.008
	*Eimeria* infected treated (Amprolium)	0.017	0.06	0.015	0.012	–	0.006	0.006	0.0019	0.005
	*Eimeria* infected treated (Toltrazuril)	–	0.0075	–	0.02	–	–	–	0.003	0.0035
7 day	Healthy	–	–	–	–	–	–	–	–	–
	*Eimeria* infected non-treated	0.005	0.0045	0.003	–	–	0.005	0.003	0.0035	–
	*Eimeria* infected treated (Amprolium)	0.007	0.027	0.007	0.0045	–	0.003	0.002	0.008	–
	*Eimeria* infected treated (Toltrazuril)	–	–	–	–	–	–	–	–	–
10 day	Healthy	–	–	–	–	–	–	–	–	– -
	*Eimeria* infected non-treated	–	–	–	–	–	–	–	–	–
	*Eimeria* infected treated (Amprolium)	–	0.005	0.003	–	–	–	–	0.002	–
	*Eimeria* infected treated (Toltrazuril)	–	–	–	–	–	–	–	–	–

Not detectable (–).

Mean ± SE (n = 3).

## 4. Discussion

Pharmacokinetic interactions between anticoccidials and antimicrobial drugs have received attention in veterinary medicine especially in the poultry industry because of their frequent use in combination. However, pharmacokinetic interactions between levofloxacin alone and with amprolium and toltrazuril have been studied in broiler chickens using the microbiological assay method for analysis [25]. Although levofloxacin and enrofloxacin are related to the fluoroquinolone group, but the results showed a significant difference and this is maybe due to a different assay method [26].

Following IV administration of enrofloxacin, the elimination half-life in control healthy broiler chickens was 6.9 ± 0.5 h which is similar to reported values in broiler chickens (6 h) by Haritova et.al and Knoll et.al. [27,28] but much shorter than values recorded previously (8.26 h) by Park et.al. [29] and (9.62 h) by Parlar et.al. [30]. Besides, the obtained values are longer than those recorded in healthy chickens (4.75 h) [31]. The recorded value of biological half-life of enrofloxacin in *Eimeria* infected chickens (5.21 h) indicated that infected birds might eliminate the drug rapidly than in control healthy chickens. Similar values were previously recorded for enrofloxacin in coryza-infected chickens (5.46 h) [32] and in *E.coli* infected broilers (3.63 h) [31]. On the other hand, the current findings showed that *Eimeria* infected birds pre-treated with toltrazuril may eliminate enrofloxacin more slowly (T_1/2λz_ 7.98 h) compared to that values in control healthy chickens. This variation may be attributed to the influence of toltrazuril on the elimination of enrofloxacin.

The obtained results showed that enrofloxacin is widely distributed in the different body compartment in healthy broiler chickens, indicated by a higher volume of distribution at steady state (Vd_ss_, 5.07 L/Kg). A similar value for Vd_ss_, (4.53 L/Kg) was determined by Dimitrova et.al. [33], but a relatively lower value was previously reported in broiler chickens for enrofloxacin (Vd_ss_ 2.7 L/Kg) [29]. This variation may be attributed to the different dosage used 10 mg/kg in the current study compared to 5 mg/kg in the other studies. On the other hand, Vd_ss_ in the current study was higher than other fluoroquinolones as levofloxacin in quails 1.25 L/Kg [34], Muscovy ducks 1.37 L/Kg [35], turkeys 1.31 L/Kg [36] and difloxacin in quails 1.54 L/Kg [37].

The value of the total body clearance determined in our study for enrofloxacin in healthy broiler (0.7 L/h^/^Kg) is nearly similar to that previously reported by Hu-GongZheng et.al. [32] in broilers but higher than that recorded (10.35 L/h/Kg) by Soliman [31]. Also, the current study showed that infection with *Eimeria* spp induced a significant increase in the value of total body clearance (1.93 L/h/Kg) in infected non-treated chickens (1.13 L. h/Kg) in infected pre-treated with amprolium and (5.76 L/h/Kg) in *Eimeria* infected pre-treated with toltrazuril. These findings explain the lower CP° recorded as a result of infection with *Eimeria* spp. A similar finding was previously recorded for enrofloxacin in *E.coli* infected broilers [31]. Furthermore, the marked decrease in Cp^0^ value recorded in *Eimeria* infected birds pre-treated with toltrazuril may explain by the rapid elimination of enrofloxacin as a result of pre-medication with toltrazuril and can be described as negative pharmacokinetic interaction.

Following oral administration, the calculated values of C_max_ and T_max_ for healthy broilers (C_max_ 2.06 µg/ml and T_max_ 1.84 h) obtained in our study were consistent with values recorded in broilers (C_max_ 1.88 µg/ml) [28] and following oral administration of enrofloxacin at 10 mg/kg (C_max_ 2.44 µg/ml and T_max_ 1.64 h) [14]. On the other hand, the obtained values were slightly higher than those reported by DaSilva et.al [38] (C_max_ 1.5 µg/ml) in broilers for enrofloxacin (10 mg/kg) and slightly lower than those reported previously in broiler chickens (C_max_ 3.82 µg/ml) [5] and in pigs (C_max_ 1.139 µg/ml) [39].

The calculated value for C_max_ (1.3 µg/ml) in *Eimeria* infected broilers pre-treated with amprolium was higher than the value determined in *Eimeria* infected non-treated birds, these findings may reflect the efficacy of amprolium against *Eimeria* infection. On the other hand, the calculated value for C_max_ (0.12 µg/ml) in *Eimeria* infected birds pre-treated with toltrazuril was very lower than values recorded for *Eimeria* infected non-treated birds (0.59 µg/ml). These findings may be attributed to rapid elimination rate constant (λz 0.34 h^−1^) coupled with pharmacological interaction previously recorded for flunixin in calves after IM administration [40] where calculated serum concentration of enrofloxacin (C_max_) was significantly lower in flunixin-treated calves.

The current results showed lower systemic bioavailability (F %) in *Eimeria* infected broilers (54.7%) compared to values of healthy birds (77.33%). Similar values for systemic bioavailability (F %) were also recorded for enrofloxacin in broilers (79.64%) [29], (80.1%) [41], (80%) [28] and (74.64%) [30]. However, our value for F% was higher than the value reported in broilers (64%) [14], (59.61%) [42] and (62.26%) [32] and was lower than the value reported in common pheasants (118%) [4].

The current results showed that enrofloxacin was found to be distributed in all tissues of healthy or *Eimeria* infected broilers. The highest concentration was detected in the liver and kidney while the lowest concentration was determined in the brain. Similar findings were previously reported for enrofloxacin in broiler chicken [28,42,43]. Besides, enrofloxacin was detected in tissues of broilers, on the 5th day after stopping of the drug medication, while for 7 days in the tissue of *Eimeria* infected birds, for 10 days in the tissue of *Eimeria* infected birds pre-treated with amprolium and only for 5 days in tissues of infected birds pre-treated with toltrazuril. These findings were consistent with the values of serum enrofloxacin concentration reported in our study. Also, the extended determination of enrofloxacin in tissues of *Eimeria* infected birds were consistent with reports for other antimicrobials under diseased conditions [42,44]. They suggested that cardiovascular changes associated with diseased condition might be enhancing extravascular distribution, be responsible for the reduced circulating concentration of drugs. The concentration of enrofloxacin in organs and tissues of broilers was higher than or equal to the corresponding serum level indicating that the penetration of enrofloxacin into these tissues which is indicated by a high volume of distribution of enrofloxacin in chickens and supported by its existence in tissues for a long time and excellence for treating urinary and respiratory tract infections caused by susceptible organisms. Similar results showing high concentrations of moxifloxacin in different tissues of chickens were reported by Goudah [45]. Pre-treatment of chickens with amprolium for 5 days before enrofloxacin administration is enough time for induction of liver microsomal CYP-450 enzymes, although Abo El-Sooud [17] found that a single dose of albendazole was sufficient to induce such effect in goats.

Fluoroquinolones have low MIC values against many Gram-negative bacteria [46]; therefore, they become effective in the treatment of gram-negative bacteria in different animal species including poultry. Scheer [47] reported that the MIC of enrofloxacin against *E.coli* was 0.008–0.06 µg/ml and Meinen et.al [48] found that MIC against *E.coli* was 0.03 µgml. The importance of maintaining plasma or tissue levels of fluoroquinolones greater than the MIC of the infecting bacteria has been demonstrated by Giguere et al. [49]. According to the clinical trials [12] proposed that the critical breakpoint determining the efficacy of quinolones is an AUC/MIC >125. On the basis of these results, oral enrofloxacin dosage of 10 mg/kg to birds gives AUC/MIC 392.67 in healthy birds, 100.33 for *Eimeria* infected non-treated bird, 155.17 for infected birds pre-treated with amprolium and 28.33 for *Eimeria* infected birds pre-treated with toltrazuril. Thus, 10 mg/kg may not be adequate dosage in *Eimeria* infected non-treated birds or pre-treated with toltrazuril, while this dosage is adequate in *Eimeria*, infected birds pre-treated with amprolium.

## 5. Conclusion

It could be concluded that *Eimeria* infection significantly decreases serum enrofloxacin concentration in broiler chickens and amprolium pre-treated-infected birds raised the decreased serum level, so the efficacy of enrofloxacin may be not affected by the concurrent administration of amprolium. In addition, serum enrofloxacin concentration of birds infected with *Eimeria* pre-treated with toltrazuril was significantly decreased than *Eimeria* infected non-treated group and accordingly, toltrazuril is adversely altering the pharmacokinetic properties of enrofloxacin. Thus, we do not recommend using both drugs concurrently.

## References

[1] Atef M, El-Banna HA, Abd El-Aty AM, et al Pharmacokinetics of difloxacin in goats. Deutschi Tierarzlt Wochensch. 2002;109(7):320–323. 12161971

[2] Atef M, Atta AH, Darwish AS, et al Pharmacokinetics aspects and tissue residues of marbofloxacin in healthy and mycoplasma gallisepticum-infected chickens. Wulfenia. 2017;24(10):80–107.

[3] James EF, Kathless P, Ann V, et al Martindale, extra pharmacopoeis. 30th ed. London: The Pharmace. Press; 1993.

[4] Lashev LD, Dimitrova DJ, Milanova A, et al Pharmacokinetics of enrofloxacin and marbofloxacin in Japanese quails and common pheasants. Br Poult Sci. 2015;56(2):255–261. 25567298 10.1080/00071668.2014.998989

[5] Kang J, Hossain MA, Park HC, et al Pharmacokinetic and pharmacodynamic integration of enrofloxacin against *Salmonella enteritidis* after administering to broiler chicken by per-oral and intravenous routes. J Vet Sci. 2019;20(2):15. 10.4142/jvs.2019.20.e15 PMC6441814 30944537

[6] Xiao X, Jiang L, Lan W, et al In vivo pharmacokinetic/pharmacodynamic modeling of enrofloxacin against *Escherichia coli* in broiler chickens. BMC Vet Res. 2018;14(1):374. 30497453 10.1186/s12917-018-1698-3 PMC6267815

[7] Anadon A, Martinez-Larranaga MR, Diaz MJ, et al Pharmacokinetic variables and tissue residues of enrofloxacin and ciprofloxacin in healthy pigs. Am J Vet Res. 1999;60:1377–1382. 10566812

[8] Broome RL, Brooks DL, Babish JO, et al Pharmacokinetic properties of enrofloxacin in rabbits. Am J Vet Res. 1991;52:1835–1841. 1664673

[9] Fathy HA, Khafallah AAW, Osman AI. Disposition kinetics of enrofloxacin (Baytril 5%) in sheep and goats following intravenous and intramuscular injection using a microbiological assay. Res Vet Sci. 2002;73:125–129. 12204629 10.1016/s0034-5288(02)00020-6

[10] Mengozzi G, Intorre L, Bertini S, et al Pharmacokinetics of enrofloxacin and its metabolite ciprofloxacin after intravenous and intramuscular administration in sheep. Am J Vet Res. 1996;57:1040–1043. 8807018

[11] Aboubakr M Evaluation of bioequivalence of two enrofloxacin formulations after intramuscular administration in goats. Korean J Vet Res. 2013;53(2):77–82.

[12] Giguere S, Sweeney RW, Belanger M Pharmacokinetics of enrofloxacin in adult horses and concentration of the drug in serum, body fluids and endometrial tissues after repeated intragastrically administrated doses. Am J Vet Res. 1996;57:1025–1030. 8807015

[13] Langston VC, Sedrich S, Boothe DM Disposition of single dose oral enrofloxacin in the horse. J Vet Pharmacol Ther. 1996;19:316–319. 8866463 10.1111/j.1365-2885.1996.tb00057.x

[14] Anadon A, Martinez-Larranga MR, Diaz MJ, et al Pharmacokinetics and residues of enrofloxacin in chickens. Am J Vet Res. 1995;56(4):501–506. 7785830

[15] Sumano LH, Gutiearrez OL, Zamora MA Bioequivalence of four preparations of enrofloxacin in poultry. J Vet Pharmacol Ther. 2001;24:309–313. 11696080 10.1046/j.1365-2885.2001.00355.x

[16] Poźniak B, Tikhomirov M, Motykiewicz-Pers K, et al The influence of age and body weight gain on enrofloxacin pharmacokinetics in turkeys-Allometric approach to dose optimization. J Vet Pharmacol Ther. 2020;43(1):67–78. 31845357 10.1111/jvp.12833

[17] Abo El-Sooud K Influence of albendazole on the disposition kinetics and milk antimicrobial equivalent activity of enrofloxacin in lactating goats. Pharmacol Res. 2003;48:389–395. 12902210 10.1016/s1043-6618(03)00179-8

[18] Greif G Immunity to coccidiosis after treatment with toltrazuril. Parasitol Res. 2000;86:787–790. 11068809 10.1007/s004360000218

[19] Greif G, Harder A, Haberkorn A Chemotherapeutic approaches to protozoa: coccidiae – current level of knowledge and outlook. Parasitol Res. 2001;87:973–975. 11728025 10.1007/s004360100403

[20] Bach U, Kalthoff V, Mundt HC, et al Parasitological and morphological findings in porcine isosporosis after treatment with symmetrical triazinones. Parasitol Res. 2003;91:27–33. 12856173 10.1007/s00436-003-0828-3

[21] Mundt H, Rödder F, Mengel H, et al Control of coccidiosis due to *Eimeria bovis* and *Eimeria zuernii* in calves with toltrazuril under field conditions in comparison with diclazuril and untreated controls. Parasitol Res. 2007;101:93–104.

[22] Ramadan A, Abo El-Sooud K, El-Bahy MM Anticoccidial efficacy of toltrazuril and halofuginone against Eimeria tenella infection in broiler chickens in Egypt. Res Vet Sci. 1997;62(2):175–178. 9243719 10.1016/s0034-5288(97)90142-9

[23] Velkers FC, Blake DP, Graat EA, et al Quantification of Eimeria acervulina in faeces of broilers: comparison of McMaster oocyst counts from 24h faecal collections and single droppings to real-time PCR from cloacal swabs. Vet Parasitol. 2010;169(1–2):1–7. 20083358 10.1016/j.vetpar.2010.01.001

[24] El-Banna HA, Gouda A, El-Zorba H Comparative bioequivalence study of three formulations of enrofloxacin in sheep. Drug Metab Letter J. 2011;5(2):85–91. 10.2174/187231211795305294 21457136

[25] El-Banna HA, El- Hewaity MH, El Latif AA Influence of amprolium and toltrazuril on the disposition kinetics of levofloxacin in broiler chickens. Egypt Acad J Biol Sci. 2013;5(2):1–10.

[26] Goudah A, Abo El-Sooud K, Shim JH, et al Characterization of the pharmacokinetic disposition of levofloxacin in stallions after intravenous and intramuscular administration. J Vet Pharmacol Ther. 2008;5:399–405. 10.1111/j.1365-2885.2008.00983.x 19000258

[27] Haritova A, Djeneva H, Lashev L, et al Pharmacokinetics and PK/PD modelling of enrofloxacin in Meleagris gallopavo and Gallus domesticus. Bulg J Vet Med. 2004;7(3):139–148.

[28] Knoll U, Glunder G, Kletzmann M Comparative study of the plasma pharmacokinetics and tissue concentrations of danofloxacin and enrofloxacin in broiler chickens. J Vet Pharmacol Ther. 1999;22:239–246. 10499235 10.1046/j.1365-2885.1999.00217.x

[29] Park SC, Yun H Bioavailability and comparative pharmacokinetics of two enrofloxacin formulations in broiler chickens. Korean J Vet Clin Med. 1997;14(2):195–200.

[30] Parlar A, Kaya S The pharmacokinetics of approved medicines including enrofloxacin in broiler. Ankara Universitiesy Veteriner Facultesi Dergisi. 2005;52(2):99–103.

[31] Soliman GA Tissue distribution kinetics of enrofloxacin in healthy and *E.coli -*infected broilers. Dtsch Tierarztl Wochenschr. 2000;107(1):23–27. 10689795

[32] Zheng H-G, Hui F-Q Pharmacokinetics of enrofloxacin and its metabolite in broilers with infectious coryza. Chin J Vet Sci. 1999;19(3):278–281.

[33] Dimitrova DJ, Lashev LD, Yanev SG, et al Pharmacokinetics of enrofloxacin and its metabolites ciprofloxacin in male and female Turkeys following intravenous and oral administration. Vet Res Commun. 2006;30(4):415–422. 16502109 10.1007/s11259-006-3303-7

[34] Aboubakr M Pharmacokinetics of levofloxacin in Japanese quails (Coturnix japonica) following intravenous and oral administration. Br Poult Sci. 2012;53(6):784–789. 23398423 10.1080/00071668.2012.745928

[35] Aboubakr M, Soliman A Comparative pharmacokinetics of levofloxacin in healthy and renal damaged muscovy ducks following intravenous and oral administration. Vet Med Int. 2014 DOI:10.1155/2014/986806 PMC3971850 24707439

[36] Aboubakr M, Uney K, Elmas M Bioavailability and pharmacokinetic profile of levofloxacin following intravenous, intramuscular and oral administration in turkeys. Br Poult Sci. 2014;55(1):115–119. 24735473 10.1080/00071668.2013.860214

[37] Aboubakr M, Elbadawy M Pharmacokinetics of difloxacin in Japanese quails (Coturnix japonica) after single intravenous and oral administration. Res Vet Sci. 2019;122:36–39. 30453178 10.1016/j.rvsc.2018.11.012

[38] DaSilva RG, Reyes FG, Sartori JR, et al Enrofloxacin assay validation and pharmacokinetics following a single oral dose in chickens. J Vet Pharmacol Ther. 2006;5:365–372. 10.1111/j.1365-2885.2006.00755.x 16958780

[39] Araneda C, Villar P, Cuadros C, et al Single and multiple pharmacokinetics of enrofloxacin and ciprofloxacin in pigs. J Bioequivalence Bioavailab. 2013;5:41–46.

[40] Abo El-Sooud K, Al-Anati L Effect of flunixin on the disposition of enrofloxacin in calves. Insight Vet Res. 2011;1:1–4.

[41] Bugyei K, Black WD, Mc-Ewen S Pharmacokinetics of enrofloxacin given by the oral, intravenous and intramuscular routes in broiler chicken. Can J Vet Res. 1999;63(3):193–200. 10480461 PMC1189547

[42] Abd El-Aziz MI, Aziza MA, Soliman FA, et al Pharmacokinetic evaluation of enrofloxacin in chickens. Br Poult Sci. 1997;38:164–168. 9158891 10.1080/00071669708417963

[43] Al-Khayyat AA, Al-Shaha OMS, Al-Khafaji BA Plasma and respiratory tissues levels of three fluoroquinolones in layer chicks. Iraqi J Vet Sci. 2000;13(1):1–16.

[44] Atef M, Youssef SAH, Amer AMM, et al Metabolic behaviour and tissue distribution of nalidixic acid in chicken. Deutschi Tierazlt Wochensch. 1991;98(8):303–306. 1935680

[45] Goudah A Pharmacokinetics and tissue residues of moxifloxacin in broiler chickens. Br Poult Sci. 2009;50(2):251–258. 19373726 10.1080/00071660802710108

[46] Prescott JF, Baggot JD Antimicrobial therapy in veterinary medicine. 2nd ed. Ames, Iowa: Iowa State University Press; 1993 p. 252–262.

[47] Scheer M Studies on the antimicrobial activity of baytril. Vet Med Rev. 1987;2:90–99.

[48] Meinen JB, Mclure JE, Rosin E Pharmacokinetics of enrofloxacin in clinically normal dogs and mice and drug pharmacodynamics in neutropenic mice with *Eschericia coli* and *Staphylococcal* infections. Am J Vet Res. 1995;56:1219–1224. 7486402

[49] Schentag JR Correlation of pharmacokinetic parameters to efficacy of antibiotics; relationships between serum concentrations, MIC value and bacterial eradication in patients with gram negative pneumonia. J Infect Dis. 1991;74:218–234. 2097710

